# Multilayer brain network modeling and dynamic analysis of juvenile myoclonic epilepsy

**DOI:** 10.3389/fnbeh.2023.1123534

**Published:** 2023-03-10

**Authors:** Ming Ke, Changliang Wang, Guangyao Liu

**Affiliations:** ^1^School of Computer and Communication, Lanzhou University of Technology, Lanzhou, China; ^2^Department of Magnetic Resonance, Lanzhou University Second Hospital, Lanzhou, China

**Keywords:** multilayer network, juvenile myoclonic epilepsy, dynamic analysis, fMRI, metrics

## Abstract

**Objective:** It is indisputable that the functional connectivity of the brain network in juvenile myoclonic epilepsy (JME) patients is abnormal. As a mathematical extension of the traditional network model, the multilayer network can fully capture the fluctuations of brain imaging data with time, and capture subtle abnormal dynamic changes. This study assumed that the dynamic structure of JME patients is abnormal and used the multilayer network framework to analyze the change brain community structure in JME patients from the perspective of dynamic analysis.

**Methods:** First, functional magnetic resonance imaging (fMRI) data were obtained from 35 JME patients and 34 healthy control subjects. In addition, the communities of the two groups were explored with the help of a multilayer network model and a multilayer community detection algorithm. Finally, differences were described by metrics that are specific to the multilayer network.

**Results:** Compared with healthy controls, JME patients had a significantly lower modularity degree of the brain network. Furthermore, from the level of the functional network, the integration of the default mode network (DMN) and visual network (VN) in JME patients showed a significantly higher trend, and the flexibility of the attention network (AN) also increased significantly. At the node level, the integration of seven nodes of the DMN was significantly increased, the integration of five nodes of the VN was significantly increased, and the flexibility of three nodes of the AN was significantly increased. Moreover, through division of the core-peripheral system, we found that the left insula and left cuneus were core regions specific to the JME group, while most of the peripheral systems specific to the JME group were distributed in the prefrontal cortex and hippocampus. Finally, we found that the flexibility of the opercular part of the inferior frontal gyrus was significantly correlated with the severity of JME symptoms.

**Conclusion:** Our findings indicate that the dynamic community structure of JME patients is indeed abnormal. These results provide a new perspective for the study of dynamic changes in communities in JME patients.

## 1. Introduction

Juvenile myoclonic epilepsy is a common epileptic syndrome that often occurs before and after puberty. It usually manifests as multiple repetitive and irregular myoclonic seizures of the bilateral or unilateral arm, accompanied by arrhythmia, and may even lead to sudden falls in patients. The main feature of JME patients is that the cortex is overexcited, which is also confirmed by neurophysiology and clinical data. It was proposed that extensive hyperconnectivity in the frontal cortex acts as the excitatory driver in the propagation of discharge (Lee et al., 2017). In addition, when the threshold of phosphene produced by stimulation of the visual cortex decreases, the visual cortex of epileptic patients can be found to be overexcited by transcranial stimulation (Brigo et al., 2013), which also explains why up to 90% of JME patients are sensitive to intermittent light stimulation (Appleton et al., 2000).

A network-based approach has been proposed to determine the pathophysiological organization of the seizure-prone state in epilepsy (Spencer, 2002). With the development of fMRI technology, resting-state fMRI has become an important technology to study the brain network of epileptic patients. At present, some resting state network abnormalities have been found in the brain network of epilepsy patients (Luo et al., 2015; Zhang et al., 2015). Furthermore, analyzing the effects of epilepsy discharges on the interaction between resting-state networks through functional network connectivity can provide a deeper understanding of epilepsy (Li et al., 2015). It can be seen that complex networks have become of major interest in epilepsy research. However, the physiological structure of the human brain is relatively complex, and it usually changes with time at rest and participates in different physiological activities. Although recording techniques such as fMRI can capture brain dynamics over time, traditional networks cannot model multiple interactions across time. A more general network framework is required to describe the evolution and interaction of the network.

As an extended model of traditional networks, multilayer networks both have the simplicity of traditional networks and are conducive to the modeling of multimodal data (Vaiana and Muldoon, 2018). In the field of neuroscience, multilayer network architecture is widely used in creating a multilayer temporal network by collecting time series data from each brain region. At present, studying the dynamic community structure of brain networks with the help of multilayer networks can not only reveal the subtle phenomena hidden in human brain dynamics but also provide powerful insights for various neurological diseases. A study on schizophrenia found that patients were more flexible than healthy controls, suggesting that the brains of patients were more disorganized during community reconfiguration (Braun et al., 2016). Multilayer directed networks were applied to the study of dynamic networks in depression and found that in the resting state, the salient network of patients with depression rarely participated in community reconfiguration (Wei et al., 2017). Patients with temporal lobe epilepsy were found to have disruptions in the dynamic network reconfiguration of the language system, and it was concluded that a multilayer network based on analysis of dynamic network reconfiguration had higher predictive accuracy for epilepsy than traditional task-based static measures (He et al., 2018). Scale scores of autism were found to be highly correlated with the flexibility of the sensorimotor network, suggesting that maintaining the stability of the motor cortex is important for normal cognitive function (Harlalka et al., 2019). Examination of abnormal dynamic network organization can provide new insights into neurological disorders and can thus contribute to further understanding of the pathogenesis of psychiatric disorders.

The multilayer temporal network model fully considers the fluctuation characteristics of brain functional connections with time and dynamically extracts the functional information of the brain network in the resting state. Abnormal dynamic network configuration has been found in some people with nervous system diseases by using multilayer temporal networks, but these dynamic characteristics are still unknown in JME patients. The cortex of JME patients is overexcited, which will affect the functional connection of the brain. Therefore, we assume that the dynamic community structure of the multilayer brain network of JME patients is abnormal.

**Figure 1:**
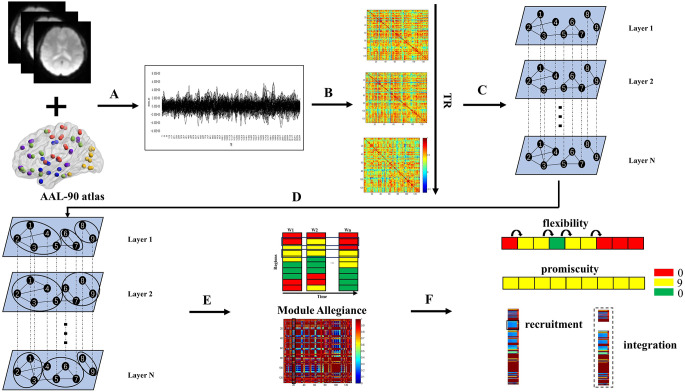
Schematic overview of the experimental approach. **(A)** Times series were extracted from AAL-90 atlas defined regions of interest using resting state functional MRI data. **(B)** A sliding window strategy (length/step = 100/2 s, 141 windows in total) was used to generate interregional coherence matrices over time. **(C)** The same nodes were connected in adjacent time slices to construct a multilayer network for each participant. **(D)** Dynamic community structure was detected by maximizing a multilayer modularity quality function. **(E)** The module allegiance matrix was calculated. **(F)** Dynamic properties were calculated.

In this study, based on the fMRI data of JME patients and healthy controls in the resting state, we employed sliding window technology to segment the collected brain imaging data of each participant, and build corresponding multilayer temporal network with intralayer connections and interlayer coupling. Next, we divide the community structures using a multilayer network community detection algorithm. Finally, the network reconstruction process was quantified by using network metrics specific to multilayer networks. The research method of this study is shown in Figure 1.

## 2. Materials and methods

### 2.1. Participants

Our study included 69 subjects, including 35 JME patients from the Epilepsy Center of Lanzhou University Second Hospital and 34 healthy subjects recruited from the local community. This study was approved by Ethics Committee of Lanzhou University Second Hospital, and all the subjects gave written informed consent after being fully informed of the study plan. The diagnosis of JME is based on the classification criteria of epilepsy in the International League Against Epilepsy (ILAE) guidelines (Engel, 2001). Patients will be excluded if they have any of the following characteristics: (1) have taken antiepileptic drugs; (2) have suffered from mental or neurological diseases; (3) suffer from developmental disorders, such as intellectual disabilities; or (4) suffer from acute diseases that affect brain scan results. The National Hospital Seizure Severity Scale (NHS3) score is usually used to measure the severity of epileptic seizures. This score is mainly related to the objective clinical events of epileptic seizures (O’Donoghue et al., 1996). It is measured in the same way as the subjective impression of epileptic patients, and needs to be completed by patients before MRI scanning. The NHS3 score is mainly composed of six factors related to epilepsy, and the score is between 1 and 23 points. The screening of the healthy control group also needs to exclude persons with febrile convulsions and seizures as well as persons with epilepsy in their families. Specific demographic characteristics are shown in Table 1.

**Table 1:** Sample demographic.

	JME	HC	*P* value
Number of subjects	35	34	-
Age (years)	16.89 ± 3.76	16.41 ± 3.09	0.472
Sex (male/female)	18/17	14/20	0.472
Duration of epilepsy (months)	39.97 ± 42.33		
NHS3 total score	8.54 ± 4.02		
Generalized convulsions	2.86 ± 1.83		
Falls	1.37 ± 1.40		
Incontinence	0.46 ± 1.15		
Loss of consciousness	1.31 ± 0.87		
Duration of recovery time	0.91 ± 0.61		
Automatisms	0.67 ± 0.96		

Continuous variables are presented as mean ± SD. Abbreviations: NHS3, national hospital seizure severity scale; JME, juvenile myoclonic epilepsy; HC, healthy controls. For continuous variables, independent sample t tests were carried out. For categorical variables, χ^2^ tests were carried out.

### 2.2. Data acquisition

Magnetic resonance images were collected on a 3T Siemens Verio scanner. During the scanning process, the subjects needed to relax, remain stable, not receive stimulation, and open their eyes to prevent the brain from participating in ideological activities. Functional images were obtained using a gradient echo echoplanar sequence. The parameters were as follows: repetition time (TR) = 2 s, echo time (TE) = 30 ms, slice thickness = 4 mm, number of slices = 33, flip angle = 90°, field of view (FOV) = 240 mm × 240 mm, matrix = 64 × 64, and number of time points = 200. Structural images were obtained using a sagittal magnetization-prepared rapid gradient echo (MP-RAGE) three-dimensional T1-weighted sequence. The parameters were as follows: TR = 1.9 s, TE = 2.99 ms, slice thickness = 0.9 mm, flip angle = 90°, FOV = 230 mm × 230 mm, and matrix = 256 × 256.

Data preprocessing was performed using GRETNA: a graph theoretical network analysis toolbox for imaging connectomes. To maintain the stability of human data during the collection process, it is necessary to delete the data of the first 10 time points, leaving 190 functional volumes for each subject. Due to the unsynchronized acquisition time of the whole head image data and the impact of head movement, slice timing correction and realignment are needed. We have removed data with movement greater than 2 mm or rotation greater than 2° caused by head movement. The remaining steps of data preprocessing are as follows: spatial normalization by DARTEL (warping individual functional images to the standard MNI space by applying the transformation matrix that can be derived from registering the final template file), spatial smoothing with a Gaussian kernel (full width at half-maximum of 6 mm), regressing out covariates (white matter, cerebral spinal fluid, and head-motion profiles were removed by multiple regression analysis to avoid noise signals), temporal linear detrending, and temporal bandpass filtering (0.01–0.1 Hz). Finally, resting-state scans were parcellated into 90 regions of interest (ROIs), using the AAL-90 atlas and time series were extracted. The AAL-90 brain atlas divides 90 brain regions into five functional networks in advance: the sensorimotor network, the visual network (VN), the attention network (AN), the default mode network (DMN), and the subcortical regions (He et al., 2009).

### 2.3. Network construction

The key to building a multilayer temporal network model is the division of time layers. To solve this problem, we mainly adopted sliding window technology. In previous dynamic network research, the minimum window size was usually set as 1/f min (f min represents the minimum frequency of the collected brain imaging data; Leonardi and Van De Ville, 2015), and the window moving step was set as one time unit (Pedersen et al., 2018; Harlalka et al., 2019; Li et al., 2019). Accordingly, we determined the window length as 100 s (50 TRs), set the step size as 2 s (1 TR) and finally divided 141 time windows. We calculated Pearson’s correlation coefficients of the 90 brain regions under each time window to build an intralayer network.

Then, we needed to couple the matrices under the adjacent time windows of the multilayer network model, where nodes and edges represented ROIs and their pairwise coherences, and layers represented the corresponding time window and were connected to each other at the same node of the adjacent time window.

As an extension of traditional networks, multilayer networks can also be represented by matrices. The matrix representation of a multilayer network is called a supra-adjacency matrix (Vaiana and Muldoon, 2018). The supra-adjacency matrix of layer M is expressed as follows:


(1) $$G=\left[\begin{array}{l} A^{\left[1\right]}\;O^{\left[1,2\right]}\;\cdots \;O^{\left[1,M_{2}\right]}\\ O^{\left[2,1\right]}\;A^{\left[2\right]}\;\cdots \;O^{\left[2,M_{2}\right]}\\ \vdots \;\vdots \;\ddots \;\vdots \\ O^{\left[M_{1},1\right]}\;O^{\left[M_{1},2\right]}\;\cdots \;A^{\left[M\right]} \end{array}\right],\left(1\leq MC_{1},M_{2}\leq M\right)$$

where A denotes the intralayer network adjacency matrix of layer M. O represents the interlayer matrix belonging to layer M_1_ and layer M_2_.

### 2.4. Dynamic community detection

For the recognition of the brain community of each subject under the multilayer network model, we mainly adopt the multilayer community detection algorithm named GenLouvain (Jutla et al., 2011). We used a generalized Louvain-like method originally developed to optimize a single-layer modularity quality function, and then extended it to optimize the following multilayer modularity quality function:


(2) $$Q=\frac{1}{2\mu }\sum_{ijlr}\left\{\left(A_{ijlr}-\gamma_{l}V_{ijl}\right)\delta_{lr}+\delta_{ij}\omega_{jlr}\right\}\delta \left(g_{il},g_{jr}\right)$$

where i and j are any two nodes and l and r are any two temporal layers. $\mu =\frac{1}{2}\sum_{jr}k_{jr}$ is the total edge weight of the network, the strength of node j in layer l is $k_{jr}=k_{jl}+c_{jl}$, the intralayer strength of node j in layer l is k_jl_, and the interlayer strength of node j in layer l is $c_{jl}=\sum_{r}\omega_{jlr\cdot }A_{ijl}$ is the edge between nodes i and j in temporal layer l. If l = r, the Kronecker delta δ_lr_ = 1 and equals 0 otherwise. Similarly, if and only if i = j, δ_lj_ = 1. The quantities g_il_ and g_jr_ represent the community assignments of node i in layer l and node j in layer r respectively, and δ(g_il_, g_jr_) is 1 if nodes belong to the same community and 0 otherwise (Bassett et al., 2013). The element V_ijl_ is the corresponding element of a null model, for which we used the Newman-Girvan null model within each layer, given by:


(3) $$V_{ijl}=\frac{k_{il}k_{jl}}{2m_{l}}$$

where $m_{l}=\frac{1}{2}\sum_{ij}A_{ijl}$ is the total edge weight in layer l. k_il_ and k_jl_ refer to the intralayer strength of nodes i and j in layer l, respectively. The parameter γ_l_ is a structural resolution parameter of layer l. The parameter ω_jlr_ is a temporal resolution parameter, giving the interlayer coupling of node j between layers l and r. There is no corresponding standard to determine the values of γ and ω. Consistent with previous study (Bassett et al., 2013), we set ω_jlr_ = ω =1 for neighbor layers, and 0 otherwise. Similarly, we set γ_l_ = *γ* = 1.

Because the GenLouvain algorithm is an extension of the Louvain algorithm, there must be uncertainty when implementing the modularization strategy, and the output results will also change after each operation. To reduce this randomness as much as possible, we carried out a multilayer community detection algorithm for each participant 50 times, and averaged the network topology metrics (recruitment coefficient, integration coefficient, flexibility coefficient, and promiscuity coefficient) obtained after these 50 runs to obtain the final result.

### 2.5. Dynamic network statistics

#### 2.5.1. Module allegiance

Module allegiance is mainly used to measure the degree to which brain regions belong to the same community (Bassett et al., 2015). An N × N square matrix (N is the number of brain regions) is usually employed to represent its results, and each element P_ij_ in the matrix represents the frequency with which nodes i and j are allocated to the same community in the whole time period. If nodes i and j are in the same community, P_ij_ is 1; otherwise, P_ij_ is 0. It can be written as follows:


(4) $$P_{ij}=\frac{1}{OT}\sum_{o=1}^{O}\sum_{t=1}^{T}\alpha_{i,j}^{k,0}$$

where O represents the total number of multilayer community detection algorithms executed, and t is the total number of time windows. If nodes I and j are assigned to the same community, $a_{i,j}^{k,o}=0$; otherwise, $a_{i,j}^{k,o}=1$.

#### 2.5.2. Recruitment and integration

Based on the results of the module allegiance matrix, we calculated the corresponding recruitment and integration coefficients. These two metrics are used to measure the probability of alliances within functional networks and across networks when brain communities are reconfigured, reflecting the ability of dynamic interactions within and across subnetworks.

The recruitment coefficient is defined as the probability of a regional alliance (within the same community) from the same functional subsystem (Mattar et al., 2015). The calculation formula of the recruitment coefficient of node i in functional network S is:


(5) $$R_{i}^{S}=\frac{1}{n_{S}}\sum_{j\in S}P_{ij}$$

where n_s_ is the number of nodes in network S.

The integration coefficient is defined as the probability of a regional alliance (within the same community) from other functional subsystems (Mattar et al., 2015). The calculation formula of the integration coefficient of node i in functional network S is:


(6) $$I_{i}^{S}=\frac{1}{N-n_{S}}\sum_{j\in S}P_{ij}$$

#### 2.5.3. Flexibility and promiscuity

Flexibility represents the probability of community allocation changes in continuous time windows (Bassett et al., 2011). The flexibility of a node i is given by:


(7) $$F_{i}=\frac{g_{i}}{L-1}$$

where g_i_ is the number of times that the node changes its community and L is the total number of strain steps.

Promiscuity represents the proportion of nodes in the network participating in all communities at least once (Papadopoulos et al., 2016). By comparing the size of promiscuity of a node, we can determine whether the node only switches back and forth between the same two communities, or truly participates in different communities. The promiscuity of a single node i is given by:


(8) $$\Psi_{i}=\frac{M_{i}-1}{Com-1}$$

where M_i_ is the number of communities in which the node has participated and Com is the total number of communities. Because promiscuity Ψ_i_ is a decimal from 0 to 1, the communities where the node is initially located are generally not considered. *Ψ* = 0 if the node participates in only one community, and *ψ* = 1 if it participates in every community in the network.

#### 2.5.4. The core-periphery system

The core-periphery system is a mesoscale structure that exists in parallel with the community. The division of the core-periphery system of the brain network is determined by the flexibility of the brain regions (Bassett et al., 2013). In this study, the 10 nodes with the lowest flexibility were considered core regions, and the 10 nodes with the highest flexibility were considered peripheral regions. The brain regions belonging to the core system are often responsible for stable functions, while the brain regions belonging to the peripheral system are mainly responsible for flexible coordination of the functions of various parts.

This research ran all the codes in MATLAB, and the solution functions of these network topology metrics (recruitment, integration, flexibility, and promiscuity) were obtained in the Network Community Toolbox^1^.

### 2.6. Statistical analysis

In this study, a dataset of independent variables for each participant included the following variables: duration of epilepsy, generalized convulsions, falls, incontinence, loss of consciousness, duration of recovery time, and automatisms. The NHS3 total scores served as dependent variables. Normal distribution was tested for each continuous variable through the Kolmogorov-Smirnov test. For normally distributed variables, we employed an independent-samples t test to test the differences between groups. For unordered categorical variables, such as sex, we used the chi-square distribution to test the differences between groups. The alpha level was set at *P* < 0.05 for all tests with appropriate correction for multiple comparisons.

This study included various types of analysis, and not all analyses had the sample sizes validated. However, in quality of community, group comparisons at the functional network level and group comparisons at the node level, the statistical power analysis was performed by GPOWER 3.1.9.7 software (Faul et al., 2007) using the following parameters: independent samples t test; two tailed; nominal significance level α= 0.05; *d* = 0.6; *n1* = 34; *n2* = 35. The sample size was demonstrated to achieve 70% power to detect differences. Please see Supplementary Materials for more detail.

## 3. Results

### 3.1. Quantity and quality of community

**Figure 2:**
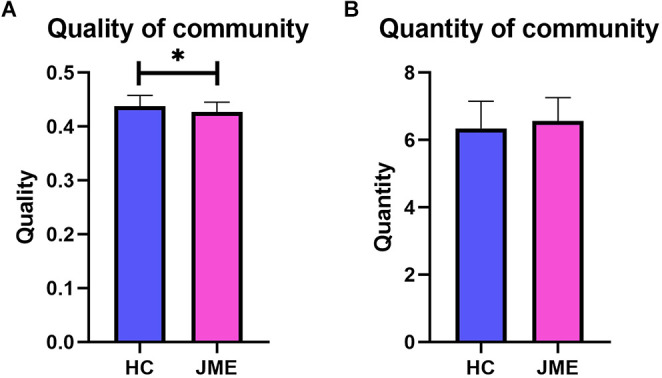
Differences between the healthy controls group and the JME patients group in quality and quantity of community. **(A)** Quality of community. **(B)** Quantity of community. Asterisks represent group differences; * denotes *P* < 0.05, FDR-corrected.

The quantity of community is the number of communities that appear in the entire time series of the multilayer network, while the quality of community is quantified by the modularity Q of the multilayer network. As shown in Figure 2, the JME group showed significantly decreased modularity Q (*t*_(67)_ = 2.335, *P*_(FDR)_ = 0.0225; Figure 2A) in the quality of community. However, there was no difference between the two groups in the quantity of community (*t*_(67)_ = 1.213, *P*_(FDR)_ = 0.2292; Figure 2B).

### 3.2. Group comparisons at the functional network level

**Figure 3:**
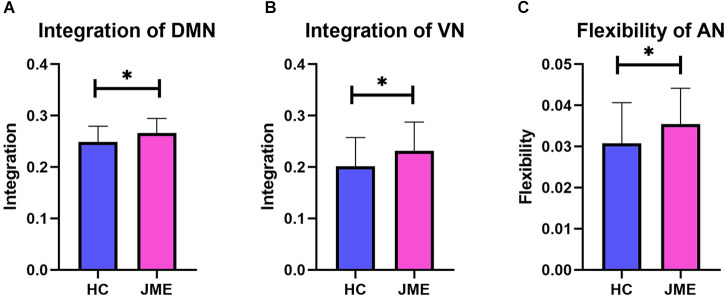
Differences in metrics results between the healthy control groups and the JME groups at functional networks level. **(A)** Integration of DMN. **(B)** Integration of VN. **(C)** Flexibility of AN. Asterisks represent group differences; * denotes *P* < 0.05, FDR-corrected.

By averaging the measurement results of all nodes in the functional network, the corresponding metric results at the functional network level are obtained. When conducting multiple comparison correction of FDR, it was found that the integration of the DMN (*t*_(67)_ = 2.402, *P*_(FDR)_ = 0.0191; Figure 3A) and VN (*t*_(67)_ = 2.226, *P*_(FDR)_ = 0.0294; Figure 3B) in the JME group showed a significant upwards trend compared to the healthy controls. In addition, the flexibility of AN (*t*_(67)_ = 2.101, *P*_(FDR)_ = 0.0394; Figure 3C) in the JME group was significantly higher than that in the healthy subjects.

### 3.3. Group comparisons at the node level

**Figure 4:**
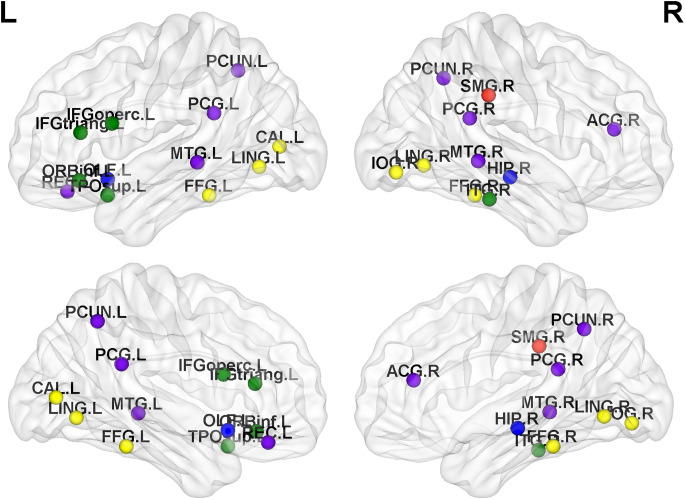
Distribution map of abnormal brain regions with significant differences in metrics (recruitment, integration, flexibility or promiscuity). Among them, the red nodes belong to the sensorimotor network, the yellow nodes belong to the VN, the green nodes belong to the AN, the blue nodes belong to the subcortical region, and the purple nodes belong to the DMN.

**Figure 5:**
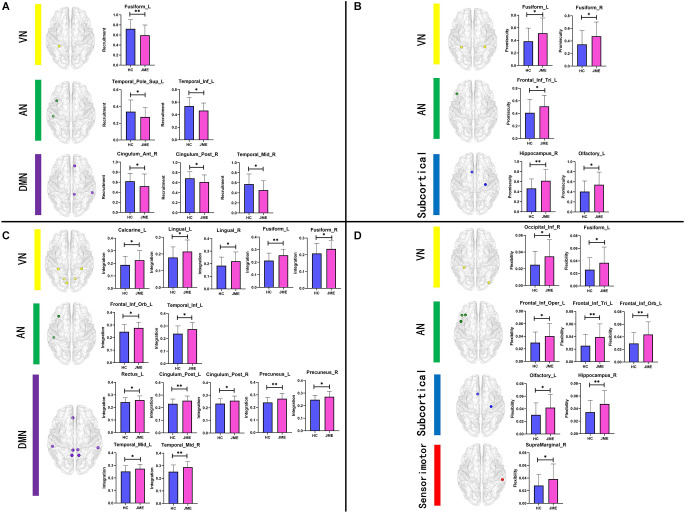
Bar charts of group differences at the node level. Brain regions with significant differences in **(A)** recruitment, **(B)** promiscuity, **(C)** integration, and **(D)** flexibility. Asterisks represent differences between groups; * denotes *P* < 0.05 and ** denotes *P* < 0.01, FDR-corrected.

By analyzing the recruitment, integration, flexibility, and promiscuity coefficients at the node level, we further identified regions of abnormal community reconstruction in the brain networks of JME patients (Tables s 2, 3 and Figure 4). For recruitment, it was found that the recruitment of six brain regions from three functional networks in the JME group showed a significant downwards trend compared to the healthy controls (Figure 5A). Specifically, there was only one region belonging to the VN, two regions belonging to the AN, and three regions belonging to the DMN. For promiscuity, it was found that the promiscuity of five brain regions from three functional networks in the JME group showed a significant upwards trend compared to the healthy controls (Figure 5B). Specifically, there were two regions belonging to the VN, just one region belonging to the AN, and two regions belonging to the subcortical regions. For integration, it was found that the integration of fourteen brain regions from three functional networks in the JME group showed a significant upwards trend compared to the healthy controls (Figure 5C). Specifically, there were five regions belonging to the VN, two regions belonging to the AN and seven regions belonging to the DMN. For flexibility, it was found that the flexibility of eight brain regions from four functional networks in the JME group showed a significant upwards trend compared to the healthy controls (Figure 5D). Specifically, there was only one region belonging to the sensorimotor network, two regions belonging to the VN, three regions belonging to the AN, and two regions belonging to the subcortical regions.

**Table 2:** Brain regions showing significant differences in recruitment and integration.

Recruitment				Integration			
Regions	ROI	Network	*P*	Regions	ROI	Network	*P*
ACG.R	32	DMN	0.0473	ORBinf.L	15	AN	0.0169
PCG.R	36	DMN	0.0272	REC.L	27	DMN	0.0489
FFG.L	55	VN	0.0076	PCG.L	35	DMN	0.0088
TPOsup.L	83	AN	0.0437	PCG.R	36	DMN	0.0113
MTG.R	86	DMN	0.0123	CAL.L	43	VN	0.0297
ITG.L	89	AN	0.0253	LING.L	47	VN	0.0306
				LING.R	48	VN	0.0415
				FFG.L	55	VN	0.0021
				FFG.R	56	VN	0.0466
				PCUN.L	67	DMN	0.003
				PCUN.R	68	DMN	0.0157
				MTG.L	85	DMN	0.0246
				MTG.R	86	DMN	0.0032
				ITG.L	89	AN	0.0104

**Table 3:** Brain regions showing significant differences in flexibility and promiscuity.

Flexibility				Promiscuity			
Regions	ROI	Network	*P*	Regions	ROI	Network	*P*
IFGoperc.L	11	AN	0.0238	IFGtriang.L	13	AN	0.0311
IFGtriang.L	13	AN	0.0238	OLF.L	21	subcortical	0.0186
ORBinf.L	15	AN	0.0026	HIP.R	38	subcortical	0.0031
OLF.L	21	subcortical	0.0222	FFG.L	55	VN	0.0215
HIP.R	38	subcortical	0.009	FFG.R	56	VN	0.0186
IOG.R	54	VN	0.0237				
FFG.L	55	VN	0.0472				
SMG.R	64	sensorimotor	0.0479				

### 3.4. Group comparisons of the core-periphery system

**Figure 6:**
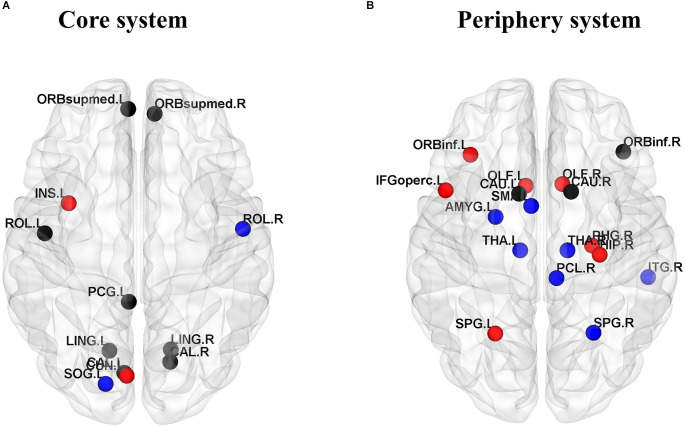
Consistent and inconsistent core-periphery system between the healthy control groups and the JME groups. **(A)** Core system. **(B)** Periphery system. Among them, the black nodes represent common nodes in two groups, the blue nodes represent nodes specific to the HC, and the red nodes represent nodes specific to the JME patients.

We divided the core-periphery system of the JME group and the healthy subject group, and compared the differences in brain regions contained in the core system and peripheral system of the two groups to further analyze the abnormal brain network organization of patients. In this study, the flexibility of each node of all subjects was averaged, the top 10 nodes with the least flexibility were regarded as the core region, and the bottom 10 nodes with the highest flexibility were regarded as the peripheral region. We observed that the left insula and left cuneus are core regions specific to the JME group (Figure 6A), and the specific peripheral structures of the JME group are mostly located in the prefrontal lobe and hippocampus (Figure 6B).

### 3.5. Correlations between abnormal nodes and NHS3 scores

**Figure 7:**
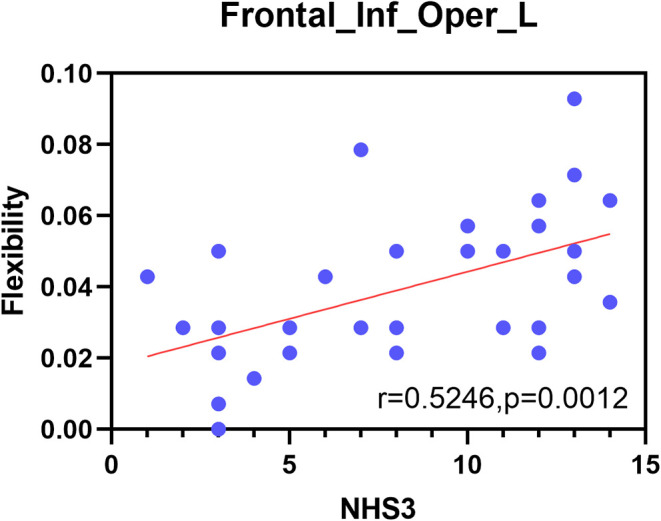
Correlation between the NHS3 scores and the opercular part of inferior frontal gyrus.

To further understand the relationships between the metrics and the severity of JME symptoms, correlations between significantly different measures and NHS3 scores were examined at the functional network and node levels in this study. Finally, no significant correlation was found at the functional network level. At the node level, however, there was a significant positive correlation between flexibility of the opercular part of the inferior frontal gyrus and the severity of JME symptoms (*r* = 0.5246, *P* = 0.0012; Figure 7).

## 4. Discussion

Previous research findings have confirmed that the cortex of JME patients has the problem of overexcitation, which undoubtedly affects the functional connection of the brain. To explore the subtle dynamic changes in brain functional connections in JME patients, different from previous static network research methods, this study applied the temporal multilayer model to patients with JME. Multilayer network community detection was carried out. By introducing statistical metrics specific to multilayer networks, such as recruitment, integration, flexibility, promiscuity, and core-peripheral systems, the change in the community structure of JME patients was explored. The results showed that compared with healthy controls, the brain network reconstruction of JME patients was abnormal. Therefore, this study provides a new perspective for research on the dynamic changes in communities in patients with JME.

Regarding community detection, compared with healthy controls, JME patients had a significantly decreased modularity. Modularity is used to measure the degree to which the network is divided into functionally independent modules, reflecting the ability of the brain to process specific functions within highly interconnected functional subnetworks (Paldino et al., 2017). Modularity can also quantify the development of the brain. Previous studies of differences in brain development related to cortical thickness have confirmed that certain brain regions tend to mature together (Garcia-Ramos et al., 2019), which explains modular behavior in the brain region. The degree of modularity affects the ability of the network to adapt to changing functional requirements (Baniqued et al., 2019). We inferred from the results of community division that the community structure of JME patients had changed.

To explore the abnormal dynamic structure of the brain network of JME patients, this study further analyzed the functional network and node level. At the functional network level, the integration of JME patients’ DMN and VN increased significantly, suggesting that brain regions in these two functional networks tend to align with brain regions in other experience functional networks during dynamic reconfiguration. The DMN often participates in the cognitive process of the brain and plays a key role, such as remembering, envisioning the future and making social inferences (Buckner and DiNicola, 2019). Recently, dysfunctions of the DMN have been observed in JME patients, such as during seizures, and the clinical symptoms of patients are often related to the coupling of spontaneous fluctuations and functional connectivity in posterior regions of the DMN (Jia et al., 2018). Furthermore, researchers have also found changes in the VN in epilepsy patients. For example, the ICA technique that detects functional brain networks in patients with temporal lobe epilepsy was found to be deficient in advanced visual function (Zhang et al., 2009). Other researchers have found a positive correlation between photoparoxysmal response grades and the severity of cortical tremors and duration of epilepsy (Wang et al., 2022). In addition, the flexibility of the AN was also significantly increased in JME patients, suggesting that the community formed by brain regions in the AN is unstable, as has been demonstrated by previous studies of epilepsy. For example, some studies have shown that the dorsal attention network of patients with mesial temporal lobe epilepsy differs from that of healthy controls (Liao et al., 2010). In addition, an analysis of resting functional connectivity in children with absence epilepsy revealed an overall decrease in AN connections, and behavior measures used to quantify inattention were significantly higher than in healthy subjects (Killory et al., 2011).

Based on the analysis at the functional network level, we focused on the differences in network metrics among the internal nodes of the DMN, VN, and AN. The study found that in the DMN network, except for the anterior cingulate and paracingulate gyrus, the integration of the remaining seven nodes was significantly increased and mainly distributed in the prefrontal lobe, posterior cingulate gyrus, precuneus, and temporal lobe. In the VN network, except for the inferior occipital gyrus, the integration of the remaining five nodes was significantly increased and mainly distributed in the occipital lobe, lingual gyrus, and fusiform gyrus. In the AN network, except for the superior temporal gyrus of the temporal pole and inferior temporal gyrus, the flexibility of the remaining three nodes was significantly increased, and they were mainly located in the frontal lobe. High integration indicates frequent interaction with brain regions in other functional networks, and high flexibility indicates unstable brain network organization. A study has shown that the frontal lobe is mainly involved in working memory (Baddeley, 1992), executive functions, and prospective memory. In addition, during ^1^H-magnetic resonance spectroscopy studies, researchers found that the concentrations of N-acetyl aspartate in the prefrontal lobes of JME patients decreased significantly, indicating abnormalities in the prefrontal cortex (Savic et al., 2004). When VBM analysis was performed in JME patients, the volume of gray matter in the frontal lobes was reduced, possibly due to preponderant discharges of the frontal lobes of JME patients (Lancman et al., 1994), resulting in reduced volume of gray matter in local neurons or neuron cell impairment (Doble, 1999). The posterior cingulate cortex, which plays a significant role in the DMN, has visuospatial and memory function, and is often seen as an important region responsible for diffusing connections during the propagation epilepsy discharge (da Silva Braga et al., 2014). There is evidence linking the cingulate cortex to structural defects and dysfunction in generalized tonic-clonic seizures (Luo et al., 2011). The precuneus participates in the epilepsy discharge network and shows obvious changes in activity within a few seconds before discharge (Bai et al., 2010). In addition, before epileptic discharge, the precuneus showed the strongest connection strength. Increased connectivity between the precuneus and the nearby cortex (Qin et al., 2020), especially in motor-related regions, has been observed, suggesting that hyperconnectivity of the precuneus may be an important trigger for epilepsy discharge (Qin et al., 2022). The temporal lobe is often affected by epileptic activity in different types of epilepsy. The study found that the left middle temporal gyrus in JME patients decreased in volume of gray matter and increased functional connectivity with the left inferior parietal lobule, right postcentral gyrus, and left superior temporal gyrus (Zhong et al., 2018), while the left temporal gyrus was often involved in auditory and visual processing streams (Binder et al., 2009). The occipital lobe is associated with photosensitive properties in JME patients, especially idiopathic occipital lobe epilepsy (Chilosi et al., 2006). Existing studies show that 30% of JME patients are photosensitive (Wolf and Goosses, 1986). Some studies have found that there are abnormal cortical thickness and gray matter volume in the occipital lobe of JME patients, and most of them occur in the fusiform, lingual gyrus, and lateral occipital cortex (Park et al., 2018). Our results support the abnormal structure and functional performance of the above nodes.

To further analyze the causes of abnormal community structure, we also need to pay attention to the nodes that are “active” but become “isolated” in JME patients. Therefore, the core-peripheral structure of the brain regions was divided for JME patients and healthy controls, with the 10 nodes with the least flexibility considered the core system and the 10 nodes with the highest flexibility considered the peripheral system. The study found that the left insula and left cuneus are core regions specific to the JME group, which has been confirmed in previous studies. For example, the betweenness centrality of the node is high, indicating that the node communicates frequently with other nodes, while the betweenness centrality of the left insula of children with Generalized Tonic-Clonic Seizures (GTCS) is significantly low (Sporns, 2011). It is inferred that the decrease in betweenness centrality in the left insula is due to epilepsy disruption of the structural pathways. Structural damage to the insula not only affects the functional pathways connected to it but also affects the motor and somatosensory function (Li et al., 2020). The cuneus is usually considered to be responsible for visual functions, and it is the center of many long-range white matter fibers to support nonvisual functions (Si et al., 2021). In addition, the specific peripheral structures of the JME group are mostly located in the prefrontal lobe and hippocampus. Previous studies have found atrophy of the hippocampus in JME patients (Tae et al., 2006), potentially due to negative effects on the production of new neurons during epileptic seizures (Kuruba et al., 2009) and hyperexcitability leading to cytotoxicity and cell death (Choi, 1994).

Finally, this study included a correlation analysis between the metrics of functional networks and nodes with significant differences and the NHS3 scores. The results showed that only the flexibility of the opercular part of the inferior frontal gyrus was significantly positively correlated with NHS3 scores, suggesting that this brain region is sensitive to the severity of JME symptoms. The opercular part of the inferior frontal gyrus is closely related to the insula in structure and function. In terms of structure, the insula is laterally covered by the opercular parts of the frontal, parietal, and temporal lobes (Türe et al., 1999). In terms of function, there are functional correlations between the insula and orbitofrontal cortex, supplementary motor area, frontal operculum, and other physiological structures (Ben et al., 2010). The insula often performs a major role in consciousness and emotion, and is also responsible for the conversion of the DMN and central executive network (Jakab et al., 2012). A study pointed out that the symptoms of emotional instability, difficulties in social adjustment and disinhibited behavior in JME patients are related to frontal-insula network activity (Frieder et al., 2015). Furthermore, in the relevant area of the spread of the early onset of insular epilepsy, it can be found that the opercular cortex and the insular lobe are highly connected (Freri et al., 2017). This is consistent with the results of our research on the abnormal dynamic network structure associated with JME diseases, providing a new perspective for research on the dynamic changes in communities in patients with JME.

In general, various functional systems will reduce dynamic interaction in the resting state of the brain and as little integrative activity as possible to maintain basic brain activity in the most efficient and cost-effective energy configuration. In JME patients, epileptic discharge caused sudden, transient disturbances in brain activity, resulting in abnormal organization of brain networks. This coincides with the results of this research that found reduced recruitment, increased integration, increased flexibility, and increased promiscuity in some functional networks and some brain regions. This confirms our research hypothesis that the dynamic community structure of JME patients is abnormal. This research also provides a new perspective for the study of dynamic communities of JME patients.

There are some limitations in this study. First, our sample size was small. Whether the results can be generalized to the general population still needs confirmation and exploration. Second, when we constructed the network, the brain template chosen was AAL-90, which removed the cerebellum regions. Previous studies have found that the pathogenesis of JME is related to the potential spontaneous activity of the cerebellum (Zhong et al., 2018). Further research is needed to address these issues in the future. Third, some studies have found that men and women have different susceptibilities to epilepsy (Savic, 2014). In the future, sex will be considered to study the differences in brain dynamics between men and women with JME.

## Date availability statement

The raw data supporting the conclusions of this article will be made available by the authors, without undue reservation.

## Ethics statement

The studies involving human participants were reviewed and approved by The Epilepsy Center of Lanzhou University Second Hospital. Written informed consent to participate in this study was provided by the participants’ legal guardian/next of kin.

## Author contributions

MK, CW, and GL designed the experiment and revised the manuscript. MK and CW wrote the manuscript. GL recorded and collected the data. CW performed the data analysis. All authors contributed to this article and approved the version submitted.

## Footnotes

^1^ http://commdetect.weebly.com/
