# Supplementary material for: Multilayer brain network modeling and dynamic analysis of juvenile myoclonic epilepsy
Source: Front Behav Neurosci. 2023 Mar 10;17:1123534. doi: 10.3389/fnbeh.2023.1123534 (PMC10036585; doi:10.3389/fnbeh.2023.1123534)
Supplement: Supplementary file 1 [file Data_Sheet_1.PDF]

Supplementary Material

Supplementary Table 1 Power Analysis

Items			Sample Size(N)	Mean(M)	Standard Deviation(S)	Effect Size(d)	Power
Quality of community		HC	34	0.437625	0.019887	0.56209	0.63337
		JME	35	0.426807	0.018583		
Integration	DMN	HC	34	0.249141	0.030242	0.57805	0.65766
		JME	35	0.266072	0.028306		
	VN	HC	34	0.201331	0.056007	0.86338	0.94222
		JME	35	0.231355	0.056018		
Flexibility	AN	HC	34	0.030824	0.009826	0.5054	0.54333
		JME	35	0.035499	0.008636		
Recruitment	ACG.R	HC	34	0.619749	0.156684	0.48817	0.5153
		JME	35	0.5195	0.244524		
	PCG.R	HC	34	0.686579	0.130545	0.54408	0.6053
		JME	35	0.613086	0.139463		
	FFG.L	HC	34	0.722377	0.18013	0.66347	0.77508
		JME	35	0.594899	0.203438		

Supplementary Table 2 Continued

Items			Sample Size(N)	Mean(M)	Standard Deviation(S)	Effect Size(d)	Power
Recruitment	TPOsup.L	HC	34	0.338037	0.139525	0.49429	0.52526
		JME	35	0.275228	0.113253		
	MTG.R	HC	34	0.573922	0.201357	0.61945	0.71753
		JME	35	0.453983	0.185563		
	ITG.L	HC	34	0.538031	0.140646	0.5503	0.61506
		JME	35	0.466014	0.1203		
Integration	ORBinf.L	HC	34	0.246664	0.056865	0.58893	0.67387
		JME	35	0.276972	0.045423		
	REC.L	HC	34	0.242454	0.034714	0.48285	0.50661
		JME	35	0.258783	0.032898		
	PCG.L	HC	34	0.230814	0.03813	0.65025	0.75852
		JME	35	0.255382	0.037432		
	PCG.R	HC	34	0.233645	0.038278	0.62681	0.72762
		JME	35	0.257182	0.036809		
	CAL.L	HC	34	0.187164	0.069836	0.5353	0.5914
		JME	35	0.225926	0.0749		
	LING.L	HC	34	0.179443	0.063006	0.53212	0.58635
		JME	35	0.214635	0.069123		

Supplementary Table 3 Continued

Items			Sample Size(N)	Mean(M)	Standard Deviation(S)	Effect Size(d)	Power
	LING.R	HC	34	0.184443	0.068659	0.50077	0.50077
		JME	35	0.220084	0.0736		
	FFG.L	HC	34	0.21255	0.060284	0.76905	0.88252
		JME	35	0.254782	0.04896		
	FFG.R	HC	34	0.208327	0.060451	0.4874	0.51404
		JME	35	0.235084	0.048714		
	PCUN.L	HC	34	0.239671	0.038382	0.74159	0.85889
		JME	35	0.269837	0.04285		
Integration	PCUN.R	HC	34	0.247325	0.034895	0.59788	0.68696
		JME	35	0.271	0.043799		
	MTG.L	HC	34	0.251412	0.045344	0.55266	0.61875
		JME	35	0.273665	0.034446		
	MTG.R	HC	34	0.252839	0.054011	0.73478	0.85256
		JME	35	0.289214	0.044545		
	ITG.L	HC	34	0.23889	0.061804	0.63446	0.73792
		JME	35	0.27509	0.051876		
Flexibility	IFGoperc.L	HC	34	0.029832	0.017001	0.55783	0.62679
		JME	35	0.040204	0.02006		

Supplementary Table 4 Continued

Items			Sample Size(N)	Mean(M)	Standard Deviation(S)	Effect Size(d)	Power
Flexibility	IFGtriang.L	HC	34	0.02542	0.018035	0.71582	0.83395
		JME	35	0.039388	0.020887		
	ORBinf.L	HC	34	0.029412	0.017564	0.75373	0.86971
		JME	35	0.043673	0.020186		
	OLF.L	HC	34	0.030462	0.018964	0.5641	0.63647
		JME	35	0.041837	0.021298		
	HIP.R	HC	34	0.034244	0.019084	0.64805	0.7557
		JME	35	0.047347	0.021294		
	IOG.R	HC	34	0.02479	0.016139	0.55815	0.62729
		JME	35	0.034898	0.019886		
	FFG.L	HC	34	0.02605	0.019011	0.48792	0.51489
		JME	35	0.036939	0.025193		
	SMG.R	HC	34	0.028151	0.017579	0.48633	0.51229
		JME	35	0.038367	0.023948		
Promiscuity	IFGtriang.L	HC	34	0.409454	0.215267	0.52944	0.58206
		JME	35	0.513095	0.174075		
	OLF.L	HC	34	0.405322	0.211172	0.58166	0.66308
		JME	35	0.540884	0.25306		

Supplementary Table 5 Continued

Items			Sample Size(N)	Mean(M)	Standard Deviation(S)	Effect Size(d)	Power
Promiscuity	HIP.R	HC	34	0.465196	0.189368	0.74016	0.85757
		JME	35	0.618231	0.222798		
	FFG.L	HC	34	0.385469	0.210494	0.56751	0.64168
		JME	35	0.514252	0.242249		
	FFG.R	HC	34	0.344993	0.221762	0.58085	0.66186
		JME	35	0.475238	0.226676		

Supplementary Table 2 Verification results of normal distribution hypothesis

Items		HC	JME
Quality of community		0.092658	0.126076
Quantity of community		0.092603	0.105594
Integration	DMN	0.118271	0.122661
	VN	0.064813	0.088718
Flexibility	AN	0.124448	0.092486
Recruitment	ACG.R	0.084829	0.137684
	PCG.R	0.086522	0.105609
	FFG.L	0.104649	0.087713
	TPOsup.L	0.071821	0.093367
	MTG.R	0.104614	0.076562
Integration	ITG.L	0.126657	0.096981
	ORBinf.L	0.116734	0.07979
	REC.L	0.088704	0.099082
	PCG.L	0.127067	0.086704
	PCG.R	0.090615	0.093677
	CAL.L	0.094943	0.096703
	LING.L	0.071771	0.099711
	LING.R	0.101454	0.109814
	FFG.L	0.090776	0.133828
	FFG.R	0.06525	0.127249

Supplementary Table 2 Continued

Items		HC	JME
Integration	PCUN.L	0.091966	0.153097
	PCUN.R	0.147505	0.130495
	MTG.L	0.09325	0.108836
	MTG.R	0.105572	0.149512
	ITG.L	0.095333	0.07898
Flexibility	IFGoperc.L	0.189451	0.176146
	IFGtriang.L	0.195352	0.112659
	ORBinf.L	0.166139	0.176984
	OLF.L	0.127944	0.138037
	HIP.R	0.131408	0.129687
	IOG.R	0.157018	0.150896
	FFG.L	0.154865	0.130003
Promiscuity	SMG.R	0.148925	0.117217
	IFGtriang.L	0.135495	0.155731
	OLF.L	0.091658	0.081411
	HIP.R	0.190558	0.135563
	FFG.L	0.119541	0.133686
	FFG.R	0.136392	0.199364
